# Craniotomy Improves Traumatic Optic Neuropathy

**DOI:** 10.7759/cureus.2835

**Published:** 2018-06-19

**Authors:** Juni EJYJ, Wen-Jeat Ang, Wan-Hazabbah WH, Yew Chin Tan

**Affiliations:** 1 Ophthalmology, Hospital Universiti Sains Malaysia, Kelantan, MYS; 2 Neurosurgery, Hospital Universiti Sains Malaysia, Kelantan, MYS

**Keywords:** traumatic optic neuropathy, bony spur, emergency craniotomy

## Abstract

Traumatic optic neuropathy (TON) is a rare devastating complication of traumatic head injury and is an ophthalmic emergency. Herein, we report a rare case of a 46-year-old gentleman who experienced severe blurring of vision, binocular diplopia, and pain over his left eye following a fall from a tree about three meters in height. Examinations revealed the visual acuity was 6/60 with a marked relative afferent pupillary defect and generalized ophthalmoplegia over his left eye. Emergency computed tomography (CT) brain and orbit showed a left frontotemporoparietal extradural hemorrhage, comminuted frontotemporoparietal and greater wing of sphenoid fracture with a bony spur impinging the lateral rectus and indirectly on the optic nerve. A diagnosis of left frontotemporoparietal bone fracture with traumatic optic neuropathy was made. An emergency left craniotomy, elevation of depressed skull fracture, and evacuation of clot was done. Postoperatively, his visual acuity showed marked improvement with visual acuity of 6/6 and all optic nerve functions were normal.

## Introduction

Traumatic optic neuropathy (TON), as the name suggests, is due to an impact, be it direct or indirect, to the optic nerve, causing partial or complete loss of function [1]. Direct injury to the area is less common due to the protection of the bony orbits [2]. We aim to report an interesting case where the traumatic optic neuropathy was caused by the greater wing of sphenoid fracture with a bony spur impinging the lateral rectus and indirectly on the optic nerve that recovered when elevation of depressed skull fracture was done. This is the first clinical case of such being reported.

## Case presentation

A 46-year-old gentleman who was well pre-morbidly was admitted to our neurosurgery department after allegedly sustaining a fall from about three meters' height while attempting to climb a tree. He landed head down on his left frontotemporal region against a rock. Immediately after the impact, he sustained loss of consciousness. He complained of severe blurring of vision when he regained consciousness five minutes later. It was associated with pain over his left eye. He was brought to the hospital six hours later.

At the time of presentation, his Glasgow Coma Scale (GCS) was 15. The visual acuity was 6/18 in the right eye and 6/60 in the left eye. A grade II relative afferent pupillary defect (RAPD) was well demonstrated in the left eye. Ocular motilities were full over the right eye. However, clinically there was a marked restriction of ocular movement in all gazes over the left eye. Light brightness and red desaturation of the left eye were reduced compared to the right eye. Intraocular pressures were 13 mmHg and 19 mmHg in the right and left eye respectively. Examination of the ocular adnexa revealed a periorbital ecchymosis and severe chemosis over the left eye. Anterior segment evaluation of both eyes was normal. There was no hyphema, iris sphincter tears or penetrating injury to the cornea, conjunctiva, or sclera of either eye. Bilateral fundoscopy showed normal optic disc and macular. 

**Figure 1:**
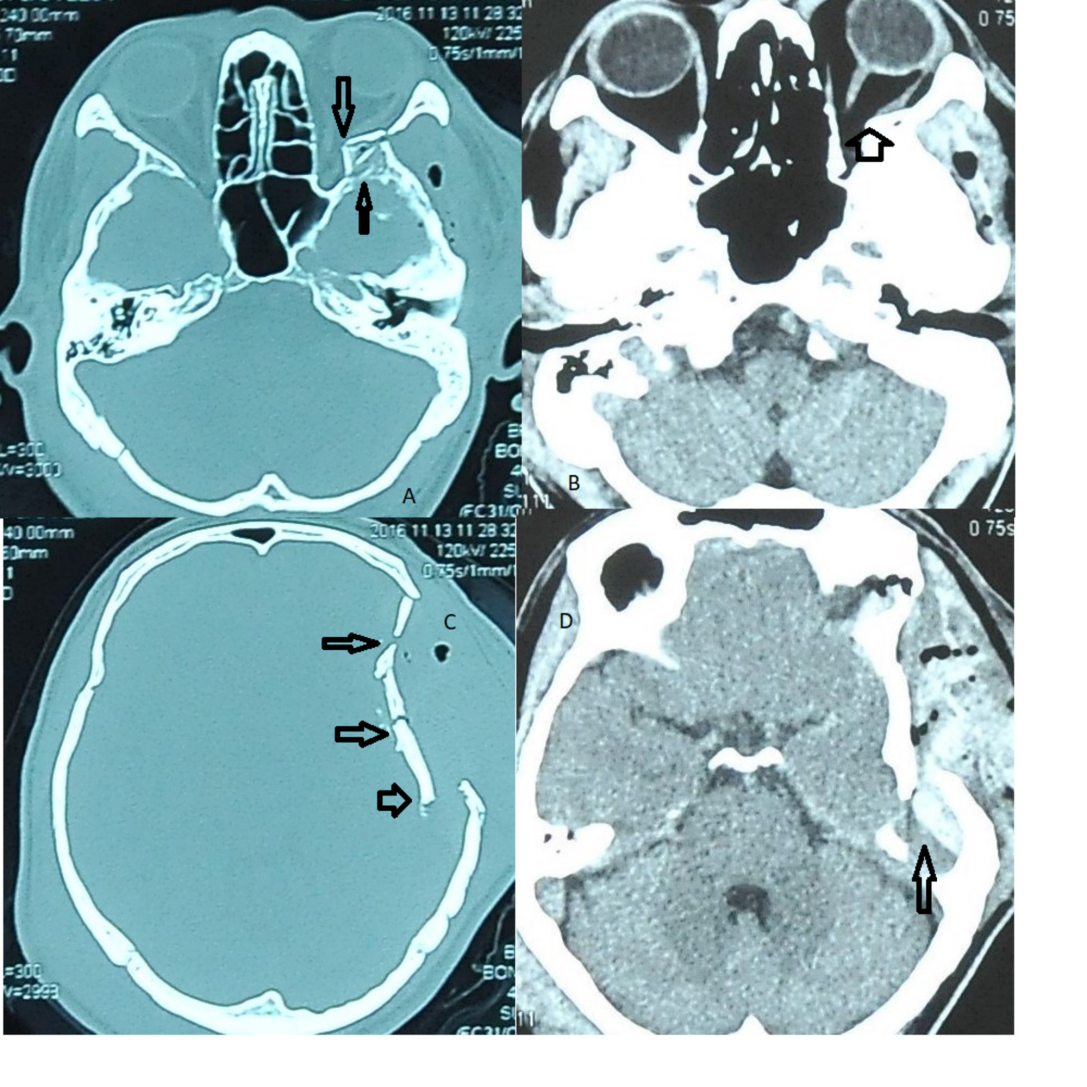
(A,B) left greater wing of sphenoid bone fracture with a bony spur abutting and impinging the left lateral rectus and indirectly the optic nerve on the same side; (C) multiple comminuted fractures in the left frontotemporoparietal; (D) left frontotemporoparietal extradural haemorrhage

An emergency computed tomography (CT) scan of brain and orbit was performed and showed a left frontotemporoparietal extradural hemorrhage with multiple comminuted fractures in the left frontotemporoparietal bone and greater wing of sphenoid fracture with a bony spur abutting and impinging the left lateral rectus and indirectly the optic nerve on the same side (Figure 1).

**Figure 2:**
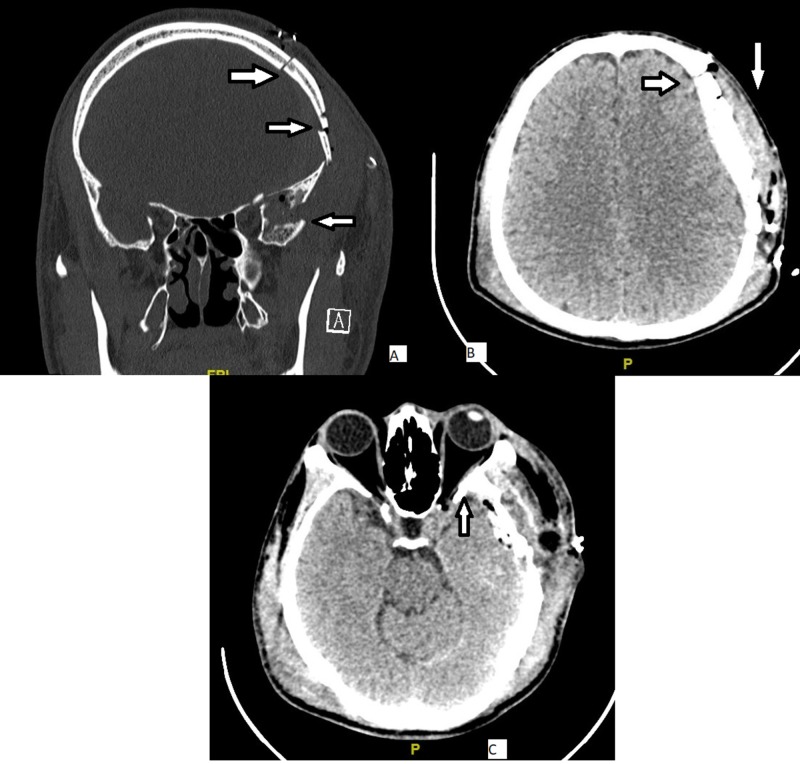
a left craniotomy was done 12 hours post trauma. (A) elevation of depressed left frontotemporoparietal skull fracture; (B) evacuation of clots; (C) bony impingement being relieved

A diagnosis of the left frontotemporoparietal skull fracture with left eye traumatic optic neuropathy (TON) was made. Being co-managed by neurosurgical team, a left craniotomy, elevation of depressed frontotemporoparietal skull fracture, and evacuation of clots was done 12 hours post-trauma (Figure 2). Intraoperatively, it was noted that the bony impingement was relieved once the depressed skull fracture was elevated. Hence, no further orbital exploration was done.

The surgery went well and was uneventful. However, the patient was not started on systemic corticosteroid in view of the intracranial bleed. Two days post-operation, his visual acuity improved to 6/6 for both eyes and the RAPD was absent. The other optic nerve function test was also normal. There was also marked improvement in his extra-ocular motility clinically. During discharge, the condition was stable. He was referred to his hometown hospital for continuation of the treatment. The follow-up six months post-operatively reveals a stable visual acuity and visual fields.

## Discussion

TON occurs in 0.5–5.0% of all closed head injuries and is a potentially blinding ophthalmic emergency [3-4] invariably leading to permanent visual loss [3]. In one report using CT imaging, about half of all TON cases were found to have an associated sphenoidal bone fracture, an indirect measure of the significant compressive forces involved at impact [3]. Direct TON is associated with penetrating wound. Indirect TON is seen in 0.5% to 5.0% of closed traumatic brain injury [4]. Visual loss is usually instantaneous; however, there have been reported cases of delayed visual loss [5].

TON refers to any insult to the optic nerve secondary to trauma. It can be classified depending on the site of injury (optic nerve head, intraorbital, intracanalicular, or intracranial) or according to the mode of injury (direct or indirect) [6]. In direct TON, there is significant anatomical disruption to the optic nerve, for example, from a projectile penetrating the orbit at high velocity or as a result of optic nerve avulsion. Indirect TON is caused by the transmission of forces to the optic nerve from a distant site without any overt damage to the surrounding tissue structures. The deformative stress transmitted to the skull from blunt trauma is concentrated in the region of the optic canal. The intracanalicular segment of the optic nerve is particularly susceptible to this form of injury because the dural sheath is tightly adherent to the periosteum at this specific location [7]. The intracranial portion of the optic nerve in close proximity to the falciform dural fold is the next most common site at risk of injury [8]. However, both direct and indirect mechanisms can contribute to optic nerve damage and a clear distinction is not always possible.

In our patient, the elevation of depressed skull surgery was done at 12 hours and the visual outcome was good. During his six month follow-up, it was shown that his visual acuity was good and remained stable. In this case, we postulate that the injury was trivial and did not cause much of an edema or any hematoma. In addition, the elevation of depressed skull fracture has indirectly relieved the body impingement. It is possible, with the relief of bony impingement, there was a subsequent reduction of perineural edema resulting in the recovery of TON and improvement of extra- ocular muscle movements. A study conducted by Thomas- Michael Wohlrab et al. found that surgical decompression of the optic nerve via the transcranial, transethmoidal, or endonasal-transethmoidal approaches within 48 hours of loss of visual acuity (or afferent pupillary defect) possibly achieves beneficial results in traumatic optic neuropathy [9]. In addition, it is also found that best visual acuity and visual fields have remained stable during a long period of follow‐up after surgical decompression [9].

The International Optic Nerve Trauma Study reported an increase in visual acuity in 32% of the surgery group, including more patients whose initial vision was non-perception to light than in the corticosteroid group or the untreated group [10]. The norm is usually to start medical decompression with intravenous methylprednisolone [11]. Surgical decompression also has some potential to give a better visual outcome [12]. However, this is the first incidence that we have come across where the elevation of a depressed skull fracture provided a good outcome for the patient.

It has been reported a visual recovery rate of 40–60% for indirect TON cases managed conservatively with baseline visual acuity being the most important predictor of final outcome. There is a significant correlation between initial and final visual acuities and patients reporting no light perception at presentation invariably have limited or no visual improvement. However, in contrary to our case, baseline visual acuity on presentation is poor; despite that, the final visual outcome is good. This could be due to an early surgical intervention which has indirectly relieved the bony impingement and subsequently decompressed the left optic nerve. Other poor prognostic factors include loss of consciousness, lack of visual recovery after 48 hours and absence of visual evoked responses. The presence of an optic canal fracture was found to predict a poor visual outcome in some. Nevertheless, this does not apply to all cases. Direct TON is a distinct category that results in severe, irreversible visual loss with little likelihood for recovery and no intervention is of proven benefit [13].

## Conclusions

TON is a condition which may cause permanent irreversible loss of vision. The main treatment options for TON include systemic corticosteroids and surgical optic nerve decompression, either alone or combination. Based on Jackson et al., [14] the variety of surgical approaches used in optic nerve decompression include intracranial, extracranial, orbital, transethmoidal, endonasal, and sublabial approaches, and selection of the technique tends to be based on the surgeon's training, background, and experience. In our patient, it has shown that prompt surgery via intracranial approaches has resulted in a reverse visual loss by alleviate the bony impingement on the optic nerve. However, at this time, conclusive evidence that surgical decompression has a beneficial role does not exist for most patients with TON. A review of the available literature, especially the International Optic Nerve Trauma Study (IONTS) and Corticosteroid Randomization After Significant Head Injury (CRASH) studies, provides insufficient evidence to include that either corticosteroid therapy and or optic canal surgery provides a therapeutic benefit over observation alone in patients with TON.
